# Locoregional treatment of *de novo* stage IV breast cancer in the era of modern oncology

**DOI:** 10.3389/fonc.2023.1083297

**Published:** 2023-01-30

**Authors:** Filippo Merloni, Michela Palleschi, Caterina Gianni, Chiara Casadei, Annalisa Curcio, Antonino Romeo, Maddalena Rocchi, Simona Cima, Marianna Sirico, Samanta Sarti, Lorenzo Cecconetto, Marita Mariotti, Giandomenico Di Menna, Ugo De Giorgi

**Affiliations:** ^1^ Department of Medical Oncology, IRCCS Istituto Romagnolo per lo Studio dei Tumori (IRST) “Dino Amadori”, Meldola, Italy; ^2^ Breast Surgery Unit, Pierantoni-Morgagni Hospital Forlì and Santa Maria delle Croci Hospital Ravenna, Forli, Italy; ^3^ Radiotherapy Unit, IRCCS Istituto Romagnolo per lo Studio dei Tumori (IRST) “Dino Amadori”, Meldola, Italy

**Keywords:** breast cancer, stage IV, primary tumor, locoregional treatment, surgery, radiotherapy

## Abstract

Approximately 6% of metastatic breast cancers arise *de novo*. While systemic therapy (ST) remains the treatment backbone as for patients with metachronous metastases, locoregional treatment (LRT) of the primary tumor remains a controversial method. The removal of the primary has an established role for palliative purposes, but it is unclear if it could also determine a survival benefit. Retrospective evidence and pre-clinical studies seem to support the removal of the primary as an effective approach to improve survival. On the other hand, most randomized evidence suggests avoiding LRT. Both retrospective and prospective studies suffer several limitations, ranging from selection bias and outdated ST to a small sample of patients. In this review we discuss available data and try to identify subgroups of patients which could benefit the most from LRT of the primary, to facilitate clinical practice decisions, and to hypothesize future studies design on this topic.

## Introduction

Approximately 6% of metastatic breast cancers (BC) arise *de novo* (1). In these patients, systemic therapy (ST), based on hormone receptor (HR) and HER2 expression, is the pillar of treatment as for patients with metachronous metastases. However, the presence of the primary tumor raises questions among clinicians about the potential benefit deriving from a local approach. Palliative removal of the primary is an established procedure as it can relieve BC patients from pain, skin ulceration, bleeding, and infections.

Surgery can also be useful to remove an ST-resistant primary tumor in presence of responsive metastatic disease.

On the other hand, it is unclear if surgery of the primary, with eventual lymph node dissection and consolidative radiotherapy, translates into a survival benefit that could justify such an invasive approach.

Pre-clinical data suggest that locoregional therapy (LRT) could be beneficial by several mechanisms. First of all, tumor burden reduction may increase CD4 and CD8 cells, improving immunologic response to cancer (2, 3). It can also minimize the dissemination of metastatic BC stem cells from the primary tumor which may act as a source of seeding (4, 5). Furthermore, some data suggest that mesenchymal stem cells released from the bone marrow may populate primary tumor more efficiently compared to metastatic sites, enhancing the metastatic potential of primary tumor cells (6).

These biological assumptions were also supported by retrospective studies that showed an association between primary tumor resection and improved survival in patients with synchronous metastases (7–10).

However, in addition to the intrinsic limitation of retrospective evidence, it is important to note that the timing of surgery is rarely specified. Patients which underwent LRT of the primary and are defined metastatic afterward because of post-operative systemic staging could have a better prognosis compared to patients who were diagnosed as metastatic before surgery. The potential influence of this stage migration bias is also outpointed by a retrospective study by Bafford et al. which highlighted a survival benefit only in those patients who underwent surgery of the primary before a diagnosis of metastatic disease (11). Consequently, randomized studies were designed to verify this hypothesis (**Table 1**).

**Table 1:** Randomized controlled trials investigating the role of locoregional treatment of the primary tumor in *de novo* stage IV breast cancer patients.

STUDY NAME	NCT	COUNTRY	ACCRUAL PERIOD	NO. OF PATIENTS	HER2-POSITIVE PATIENTS	HR-POSITIVE PATIENTS	RANDOMIZATION	SURGERY TIMING	BCS RATE	RT	MDT	BONE-ONLY MTS	PRIMARY ENDPOINT	RESULTS
ECOG-ACRIN 2108 (12)	NCT01242800	United States	2011-2025	256	LRT arm - 33% ST arm - 32%	LRT arm - 58% ST arm - 61%	After ST	After ST	25%	As per standard of early BC	Permitted	LRT arm - 44% ST arm - 39%	OS	No significant benefit
Tata Memorial (13)	NCT0019377	India	2005-2012	350	LRT arm - 26% ST arm - 35%	LRT arm - 59% ST arm - 60%	After ST*	After ST	23%	As per standard of early BC	Not specified	LRT arm - 29% ST arm - 28%	OS	No significant benefit
ABCSG – 28 POSYTIVE (14)	NCT01015625	Austria	2010-2019	95	LRT arm - 27% ST arm - 18%	LRT arm - 62% ST arm - 67%	Before ST	Before ST	29%	As per standard of early BC	Permitted	LRT arm - 49% ST arm - 36%	OS	No significant benefit
MF07-01 (15)	NCT00557986	Turkey	2008-2012	278	LRT arm - 30% ST arm - 31%	LRT arm – 85% ST arm – 72%	Before ST	Before ST	26%	As per standard of early BC	Permitted	LRT arm - 51% ST arm - 40%	OS	mOS 46 months vs 35 months (HR 0.7, p=0.0003)

HR, hormone receptor; BC, breast cancer; BCS, breast conserving surgery; RT, radiotherapy; MDT, metastases-directed treatment; MTS, metastases; ST, systemic therapy; LRT, locoregional treatment; OS, overall survival; mOS, median overall survival.

*In presence of resectable primary tumor eligible for endocrine therapy the assignment was conducted upfront.

## Evidence from randomized trials

The most recent published study which investigated the impact of primary surgery on survival is the ECOG-ACRIN 2108. A total of 256 patients with metastatic BC who did not progress during 4-8 months of ST were assigned (from February 2011 to July 2015) to LRT of the primary plus ST or ST-only continuation. Overall Survival (OS) was chosen as the primary endpoint. The statistical analysis showed no difference in 3-year OS (68.4% vs 67.9%) (HR, 1.11; 90% CI, 0.82–1.52; p=0.57). No progression-free survival (PFS) difference was observed either; only locoregional progression was reduced in the LRT group (3-year rate: 16.3% v 39.8%; P < 0.001).

Subset analysis based on HR and HER2 status did not show any subgroup which benefited from the locoregional approach (16).

An open-label randomized controlled trial with a similar study design, conducted in Mumbai, compared LRT of the primary plus ST vs ST alone in a population of stage IV BC patients with *de novo* disease.

Patients with unresectable primary underwent chemotherapy before randomization while, in presence of a resectable primary tumor eligible for endocrine therapy, the assignment was conducted upfront. A total of 350 patients were randomized. The primary endpoint was OS as for the previous study. Even in this case, no statistically different median overall survival (mOS) was reported between the two groups: 19.2 months for the LRT group vs 20.5 months in the ST alone group (HR 1.04, 95% CI 0·81-1·34; p=0·79) (13).

However, it is worth noting that the reported mOS values were considerably lower in comparison to the previous trial, in which the mOS was about 55% in both groups (16). This discrepancy can be justified by the lack of tailored therapy in this Indian trial, such as HER2-directed therapy for HER-2 positive patients and endocrine therapy for HR-positive subtypes.

The ABCSG-28 POSYTIVE trial is another phase 3 randomized study with negative results but a different design. The random assignment of *de novo* stage IV BC patients was performed before ST and patients assigned to the intervention arm underwent upfront surgery followed by ST. Only 95 patients were included between 2011 and 2015. The mOS (primary endpoint) in the surgery plus ST arm was consistently lower compared to the ST-only arm (34.6 months vs 54.8 months, HR=0.0691, p=0.267). Whilst cT3 and cN2 tumors were more represented in the surgery arm (22.2% vs 6.7% and 15.6% vs 4.4% respectively), the two groups were balanced in relation to the ST schedule.

Even if the results of this trial seem unequivocal, it must be addressed that this study was stopped early due to poor recruitment, with consequently very low statistical power, and the control arm (ST alone) performed better than expected (54.8 months vs 24 months) (14).

The Turkish Federation’s MF07-01 trial is the unique randomized study that showed a survival benefit in favor of LRT.

Similarly to the ABCSG-28 POSYTIVE study, patients were randomized to upfront surgery followed by ST or ST alone. The statistical analysis demonstrated a benefit in OS at the median 40-months follow-up which was confirmed at 10-year follow-up: mOS for the LRT arm and ST-only arm was respectively 46 months and 35 months (HR 0.7, p=0.0003). However, the two groups were unbalanced for the BC subtype, as HR-positive disease was more represented in the LRT (86% vs 73%), and the control arm included more triple negative BC (18% vs 7%) (15, 17).

These data seem to rule out a potential role for LRT of the primary in *de novo* stage IV BC patients given that the majority of randomized studies did not show a survival benefit. However, these trials are not free from inherent limits, are heterogeneous and, last but not least, there are subgroups of patients which deserve in-depth analysis.

## Oligometastatic vs polymetastatic disease

The oligometastatic disease is defined by the presence of no more than five metastatic lesions, assessed with high-resolution imaging and safely treatable with metastases-directed therapy (18).

The hypothesis that metastases-directed treatment (MDT) in oligometastatic disease could be beneficial is supported by retrospective and prospective data which showed long-term survival (19). The available randomized data rely only on two studies with conflicting results (20, 21). Waiting for data from numerous ongoing randomized trials, the current practice is to discuss oligometastatic BC patients in a multidisciplinary setting.

The chance to achieve long-term survival in oligometastatic BC patients legitimates an aggressive approach aimed at eradicating the detectable disease, making this subgroup of patients a suitable candidate for the surgery of the primary in case of *de novo* presentation.

Unfortunately, literature data regarding the survival impact of surgical resection of the primary in oligometastatic BC patients is lacking. This is probably due to the main use of LRT of the primary in clinical practice which is palliation.

In the ECOG-ACRIN 2108 trial, no survival difference was reported for oligometastatic patients (HR, 1.18; 95% CI, 0.38 to 3.67) which represented 16.3% of the study population (16).

A Similar result was shown in the Indian randomized trial in which 25% of patients had less than four metastases and were balanced between the intervention and control arm (13).

In the MF07-01 trial, the only randomized trial showing a survival benefit deriving from LRT, there was no clear distinction between oligometastatic and polymetastatic disease but no survival benefit for patients with solitary lung/liver metastases was reported for those treated with LRT, probably due to the poor representation of this subgroup (15).

However, when assessing the impact of local treatment of the primary in oligometastatic BC we cannot ignore if the limited metastases were treated with MDT. The aforementioned randomized trials generally did not specify this information, but it can be noted that MDT was generally permitted in accordance with clinical practice. In the Turkish trial it is only mentioned that irradiation rates and surgical interventions to metastatic sites were similar among the two arms (17).

On the other hand, even if the majority of randomized trials investigating MDT impact in oligometastatic BC does not include patients with uncontrolled primary (22–24), there are some exceptions (25, 26) in which it does not constitute an exclusion criterion if accessible to curative-intent treatment.

If the population of oligometastatic BC patients with synchronous metastases will be properly represented in these trials, we may have some insight into the potential survival benefit deriving from the combination of LRT of the primary and MDT with eradication intent.

## Bone-only disease

Metastatic BC patients with bone-only disease have an excellent prognosis compared to those with visceral involvement, showing an mOS that can exceed 5 years after the detection of the metastases (27, 28), thus prompting clinicians to consider the possibility of primary tumor surgery during the therapeutic process.

The BOMET MF 14-01 is a prospective multicenter registry study that evaluated the role of LRT of the primary tumor in addition to ST in *de novo* stage IV BC patients with bone-only metastases. This study included 505 patients and highlighted a prolonged survival in the median 3-year follow-up in favor of LRT of the primary (HR 0.40, p<0.0001). At 34-months median follow-up, 85 (35.4%) patients in the ST-only group and 28 (10.5%) in the LRT group died (29).

The potential survival benefit of LRT is also suggested by retrospective evidence (30–32).

In a large cohort retrospective study including 3956 BC patients with bone metastases, surgery of the primary tumor in addition to ST significantly improved OS with a median survival of 50 months versus 31 months in ST-only patients (p<0.001) (33).

Regarding randomized trials, in the Turkish study, 51% and 40% of patients presented bone-only metastases in the LRT group and ST group respectively. Notably, 23% and 15% of patients had solitary bone metastasis in the LRT and ST groups respectively. At unplanned subgroup analysis patients with solitary bone, metastasis showed a lower risk of death if treated with LRT in addition to ST (15).

Conversely, in the ECOG-ACRIN 2108 trial, which did not demonstrate any benefit of LRT in addition to ST, patients with bone-only disease (37.7%) were less represented (12).

Even if available data are not enough to conclude that LRT of the primary tumor is beneficial among patients with bone-only disease, we can affirm that this population deserves more focus.

The STEREO-OS trial, which is aimed to demonstrate that Stereotactic Body Radiotherapy of the metastases can improve survival in patients with 1 to 3 bone metastases, will also include patients with a primary tumor accessible to curative-intent treatment and might provide some information in this regard.

## What locoregional treatment modality should we prefer?

As previously mentioned, the rationale behind LRT of the primary tumor includes the reduction of tumor burden and the removal of cancer stem cells which may propagate the disease (7).

This implies that a complete removal of locoregional disease could be of utmost importance to achieve the best survival benefit, justifying surgery with clear margins and excision of involved axillary nodes.

In a retrospective study conducted by Rapiti et al. showing a survival benefit in metastatic BC patients treated with surgery of the primary, women with positive surgical margins exhibited the same survival as non-surgery ones (32).

The presence of free margins was generally associated with better survival in retrospective studies, while no clear difference was found between mastectomy and breast-conserving surgery (34–36).

Similarly, BC patients with synchronous metastases seem to benefit from axillary dissection in presence of nodal involvement even though evidence on this topic is lacking (32, 34).

As for surgery with clean margins and axillary dissection for patients with nodal metastases, local radiotherapy represents an important method in the pursuit of complete removal of locoregional disease in stage IV BC patients, considering its role in local relapse prevention and mortality reduction in early BC setting (37).

Some retrospective evidence pointed out that the omission of radiotherapy was associated with worse survival (36).

Looking at randomized studies, in the negative study published in 2022 by Khan et al., LRT consisted of surgery and radiotherapy as per standard of early-stage BC. Radiotherapy use followed NCCN guidelines and axillary dissection was reserved for patients with involved lymph nodes.

Among 107 patients which underwent surgery, 75 (70.1%) received mastectomy and 32 (29.9%) breast-conserving surgery. Radiotherapy has been employed in 44 patients (58.7%) after mastectomy and in 27 patients (84.4%) after breast-conserving surgery.

Notably, of 125 patients randomly assigned to the LRT arm, 18 (14.4%) did not receive it for various reasons, ranging from physician advice to progressive disease.

Furthermore, of 131 patients assigned to the ST-only arm, 22 (16.8%) received surgery, which was permitted for palliation, with postoperative radiotherapy in 10 cases (12). This displacement raises concerns about the negative result of the trial. LRT for primary tumor consisted of mastectomy or breast-conserving surgery with eventual postoperative radiotherapy also in the other three randomized trials.

In the Turkish trial 102 patients (74%) underwent a mastectomy, 36 (26%) breast-conserving surgery and the majority of patients received axillary dissection (92.8%) (15).

Given that also the timing of LRT could influence the outcomes, it is worth noting that in the ECOG-ACRIN 2108 trial surgery was carried out after a period of ST, while in the Turkish and ABCSG-28 POSYTIVE trials it was performed upfront.

Thus, considering the OS benefit reported in the Turkish trial (17), it might be thought that upfront surgery could provide some advantage over delayed one. In addition, this hypothesis is in accordance with previously reported biological assumptions. An upfront LRT could be convenient as it can stop the dissemination of metastatic BC stem cells from the primary earlier in the disease course (4, 5).

Surgery was performed upfront also in the ABCSG-28 POSYTIVE trial and no survival benefit for LRT was reported. However, it must be considered that this study was underpowered as it was stopped early due to poor recruitment (14).

## Biological subtypes

It is well known that, between BC subtypes, HR-positive tumors feature the best prognosis (38). As HR-positive disease tends to progress with more indolence, it is not uncommon to consider primary surgery in *de novo* stage IV patients in clinical practice.

In confirmation of this trend, HR-positive *de novo* stage IV BC patients demonstrated to benefit the most from LRT in retrospective studies (11, 39–41).

Some retrospective evidence seems also to support the use of LRT in HER2-positive subtype (7, 40, 42, 43).

In the randomized trial by Soran et al., 86% of patients were HR-positive, 30% HER2-positive, and 7% triple negative in the LRT arm, while in the ST-only arm, 73% were HR-positive, 28% HER-2 positive, and 18% triple negative.

The imbalance in biological subtypes distribution, with aggressive ones being more represented in the ST-only arm, questions the positive result of this trial.

However, in accordance with retrospective evidence, an unplanned subgroup analysis showed a benefit in OS for HR-positive patients (15).

The exploratory *post hoc* subgroup analyses of the ECOG-ACRIN 2108 trial, which was well balanced for disease subtype distribution, reported similar results across all the subgroups except for disease subtype: LRT was clearly unfavorable for triple negative patients (HR 3.33) (12).

Based on this data, HR-positive BC seems to be the best candidate for LRT in presence of synchronous metastases, while for triple-negative tumors primary surgery could be even detrimental. Any opinion on HER2-positive patients must be weighed with caution as HER2-directed therapy was not used with the same frequency in these studies.

In addition, we should consider that the usual classification of BC subtypes is being revolutionized due to the introduction of HER2-low subtype, which is forcing us to reconsider the treatment approach in every setting of BC (44).

## Modern therapy implications

ST for metastatic BC patients has dramatically evolved over the last twenty years for every disease subtype.

The recent introduction of cyclin-dependent kinase 4/6 inhibitors for metastatic HR-positive BC treatment has carried to PFS and OS improvement, further ameliorating the prognosis of this indolent subgroup (45).

Even if HER2 expression results in a more aggressive disease with a poor prognosis, the use of HER2-targeted therapy led to outstanding survival benefit in these patients. In particular, the combination of trastuzumab, pertuzumab, and docetaxel increased the number of HER2-positive long survivors with an 8-year survival rate of 37% for patients treated with dual HER-2 blockade therapy (46).

The breakthrough of antibody-drug conjugates is the best example of modern ST progress. Trastuzumab deruxtecan is changing the treatment paradigm of both HER2-positive (47, 48) and HER2-low (48) disease and sacituzumab govitecan are improving triple negative and HR-positive BC survival (49).

However, most retrospective and prospective studies investigating the role of LRT of the primary tumor in stage IV BC included patients treated with outdated ST.

The example of the open-label randomized trial conducted at Tata Memorial Hospital in India is explicative. In this study, anti-HER2 therapy was omitted in approximately 92% of HER2-positive patients (13).

On one hand, LRT of the primary tumor and modern ST seem the perfect partners for an aggressive approach aimed at eradicating the disease and reaching long-term survival. On the other hand, the development of highly effective systemic drugs may mitigate the benefits of primary tumor surgery, thus making it useless for survival benefit improvement. It is also possible that both hypotheses are true, but for different groups of patients.

The association of LRT and ST could also have a synergistic effect. Immune checkpoint inhibitors, which boost the immune response against cancer cells by targeting either programmed death 1 (PD-1) and programmed death ligand 1 (PD-L1), are establishing themselves in triple-negative disease, becoming the first line of therapy in association with chemotherapy in case PD-L1 positive disease (50, 51).

Pre-clinical data suggest that tumor promotes metastasis by systemic inflammation and cytotoxic CD8+ T cell effector function suppression (52). At the same time surgery of the primary tumor led to the rebound of antibody and cell-mediated response, restoring immunocompetence and increasing CD4 and CD8 cells in mice with metastatic BC (2, 3). Consequently, the combination of immune checkpoint inhibitors and LRT of the primary tumor could determine a strong immune response with enhanced tumor response.

## Final considerations

Retrospective studies seem consistent in supporting LRT for *de novo* stage IV BC patients (**Table 2**). However, retrospective data suffer from several limitations. The selection bias is one of the most relevant as patients who were candidates for LRT was younger, had better access to care, and a lower burden of disease (53). In addition, it is also plausible that these patients underwent more aggressive ST, thus unbalancing survival outcomes (54).

**Table 2:** Main Retrospective studies and outcome in locoregional treatment of the primary tumor in the novo stage IV breast cancer patients.

STUDY NAME	ACCRUAL PERIOD	NO. OF PATIENTS	LRT Vs No LRT	BONE-ONLY MTS	PRIMARY ENDPOINT	RESULTS
SEER (2010-2016) (33)	2010-2016	3956	Surgery Group arm – 82% Not Surgery Group– 18%	All	OS	mOS 50 months in Surgery group VS 31 months in Non-Surgery Group (p <0.001)
Geneva Cancer Registry (32)	1977-1996	300	NA	145	OS	HR=0.6, P= 0.046 (Surgery of Primary Tumor and negative margins)
French Epidemiological Strategy and Medical Economics MBC database	2008-2014	4276	LRT arm – 77.2%	2556 (40%)	OS	HR 0.65 (0.55-0.76) p 0.0001
BOMET MF 14-01 (29)	NA	505	LRT 52.5% No LRT 47.5%	All	OS	HR 0.40 (0.30-0.54) p <0.0001
SEER database (10)	1988-2011	29916	Surgery Group- 51% Not Surgery Group - 49%	NA	OS	mOS 34 months vs 18 months (HR 0.7, p=0.0003)
SEER database (8)	1988-2003	9734	Surgery Group 47% Not Surgery Group 53%	NA	OS	mOS 36 months vs 21 months (p <0.001)
Blanchard KD et al (42)	NA	395	LRT 61.3% No LRT 38.7%	NA	OS	OS 27.1 vs 16.8 (p <0.0001)

LRT, locoregional treatment; MTS, metastases; OS, overall survival; mOS, median overall survival; NA, not applicable; HR, hazard ratio.

Even though some preclinical data provide a rationale for LRT of the primary there are also concerns about the possibility that surgery may lead to cancer cells shedding into the circulation (55), a hypothesis that seems consistent with the increased incidence of distant metastases in patients which underwent LRT, highlighted in the randomized trial by Badwe et al. (13).

Randomized trials did not support LRT of the primary altogether, as confirmed by a metanalysis by Reinhorm et al. (56), but, as previously discussed, they suffer major limitations as well, ranging from outdated ST to a small sample of patients.

We must take these results with caution, and we must not label LRT of the primary as a pointless technique, also considering that advances in ST and radiotherapy/surgery methodic require continuous testing of the possible benefit deriving from LRT in stage IV BC.

**Figure 1:**
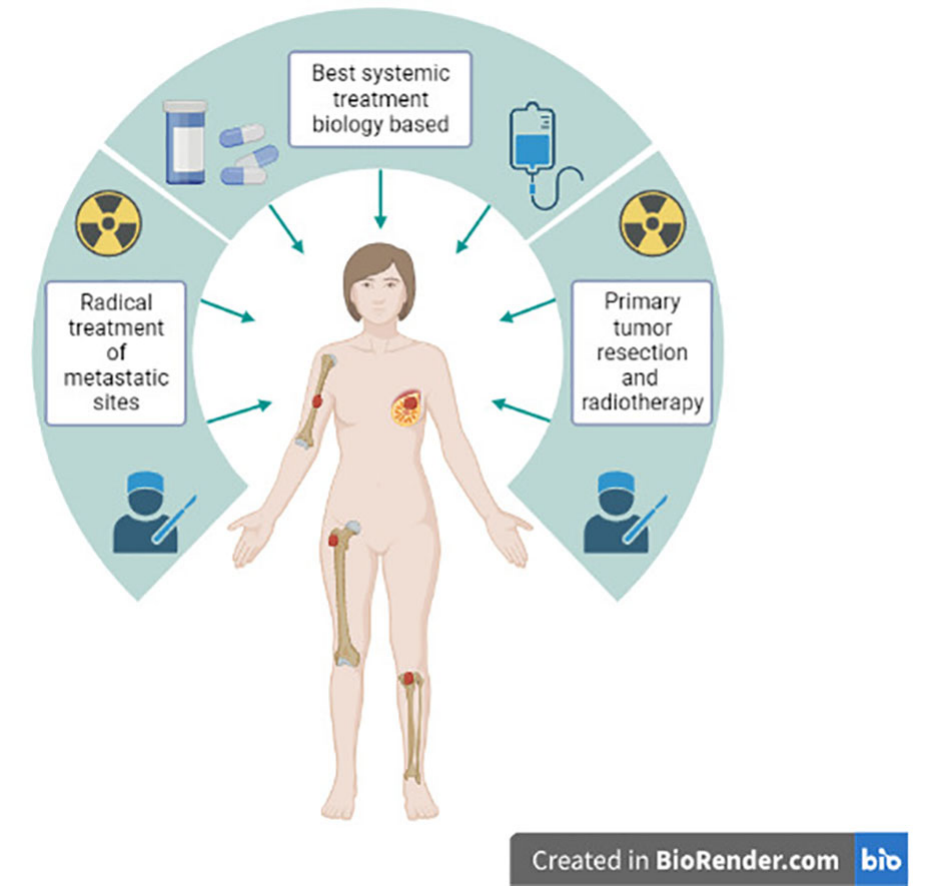
Aggressive approach combining modern systemic therapy, locoregional treatment of the primary tumor and metastases-directed therapy in stage IV *de novo* oligometastatic breast cancer patients.

We should identify the best candidate for LRT and design randomized trials accordingly. Based on the retrospective evidence and the randomized Turkish trial, oligometastatic patients, with bone-only disease and HR-positive disease could be the best candidates for studies investigating the role of LRT in stage IV BC. Regarding oligometastatic patients, the combination of LRT of the primary and metastases-directed therapy, aimed at complete eradication of detectable disease, should be investigated. This aggressive approach in combination with highly effective modern ST could provide long-term survival and, in some cases, even the cure for metastatic BC patients (**Figure 1**).

The best timing for LRT of the primary remains an issue. Upfront surgery might be the correct approach according to the potential role of metastatic BC stem cells dissemination from the primary and the significant OS benefit observed in the randomized trial by Soran et al., in which metastatic BC patients underwent upfront surgery (4, 5, 15).

On the other hand, upfront surgery could represent an overtreatment for those patients destined to progress early in the disease course.

In this context, biomarkers, such as circulating tumor cells (CTCs) and circulating tumor DNA (ctDNA), could help us characterize the metastatic disease.

In a retrospective analysis including 2436 patients with stage IV BC, a CTCs threshold of 5 cells per 7.5 ml was able to differentiate aggressive from indolent metastatic disease (57). ctDNA percentages were correlated with prognosis as well, with high levels being associated with shorter OS (58, 59).

Metastatic BC patients with high CTCs or ctDNA levels could be at higher risk of fast disease progression and, consequently, the rationale behind LRT of the primary tumor in those patients might be invalidated. Thus, the implementation of these biomarkers for patients’ stratification in future studies is suitable.

Results of two randomized trials investigating the role of LRT in *de novo* stage IV BC are awaited (60, 61). In addition, a single-arm trial investigating the role of palbociclib and LRT combination in HR-positive/HER2-negative metastatic BC is still recruiting (62).

## Conclusions

The purpose of our review is to underline the limitations and strengths of LRT of the primary tumor, to design future randomized trials, more precisely and accurately. The design of new randomized clinical trials should include modern ST, a properly selected population, and new biomarkers are strongly encouraged.

Meanwhile, in the absence of robust evidence, LRT of the primary tumor should be discussed in a multidisciplinary context for every patient with *de novo* stage IV BC

## Author contributions

All authors listed have made a substantial, direct, and intellectual contribution to the work and approved it for publication.
